# Benchmarking Domain Adaptation Methods on Aerial Datasets

**DOI:** 10.3390/s21238070

**Published:** 2021-12-02

**Authors:** Navya Nagananda, Abu Md Niamul Taufique, Raaga Madappa, Chowdhury Sadman Jahan, Breton Minnehan, Todd Rovito, Andreas Savakis

**Affiliations:** 1 Department of Computer Engineering, Rochester Institute of Technology, Rochester, NY 14623, USA; nn3264@g.rit.edu (N.N.); at7133@g.rit.edu (A.M.N.T.); rm4600@g.rit.edu (R.M.); sj4654@g.rit.edu (C.S.J.); 2 Air Force Research Laboratory, Wright-Patterson Air Force Base, OH 45433, USA; breton.minnehan.1@afresearchlab.com (B.M.); todd.rovito@afresearchlab.com (T.R.)

**Keywords:** domain adaptation, aerial datasets, unsupervised learning, visualization, deep neural networks

## Abstract

Deep learning grew in importance in recent years due to its versatility and excellent performance on supervised classification tasks. A core assumption for such supervised approaches is that the training and testing data are drawn from the same underlying data distribution. This may not always be the case, and in such cases, the performance of the model is degraded. Domain adaptation aims to overcome the domain shift between the source domain used for training and the target domain data used for testing. Unsupervised domain adaptation deals with situations where the network is trained on labeled data from the source domain and unlabeled data from the target domain with the goal of performing well on the target domain data at the time of deployment. In this study, we overview seven state-of-the-art unsupervised domain adaptation models based on deep learning and benchmark their performance on three new domain adaptation datasets created from publicly available aerial datasets. We believe this is the first study on benchmarking domain adaptation methods for aerial data. In addition to reporting classification performance for the different domain adaptation models, we present t-SNE visualizations that illustrate the benefits of the adaptation process.

## 1. Introduction

Deep neural networks were used for a variety of tasks such as image classification, segmentation, image generation, and speech recognition [1,2]. These models require a lot of labeled training data to make them generalizable and highly scalable [3,4,5]. There are practical scenarios where labeled data from the test domain are scarce or not available, and supervised methods don’t maintain their performance, especially when the training and test data are drawn from different distributions. An example of this would be to deploy a model trained on images from one sensor corresponding to the source domain, and test on images obtained from another sensor corresponding to the target domain. We expect a drop in the accuracy due to the differences between the two domains [5], and thus domain adaptation (DA) algorithms are necessary to recover performance. A common application of domain adaptation is the case of unsupervised domain adaptation (UDA), where there are labeled source data for training but unlabeled target data [4,6]. DA can reduce the need for costly labeling of the target domain data by adapting the labels from the source domain [7].

This benchmarking study focuses on unsupervised DA methods for classification without access to any labeled target domain data. Several surveys were conducted on the topic of domain adaptation [5,7,8,9,10,11,12,13,14] or transfer learning where the target domain data are labeled [15,16,17,18,19,20]. All of the DA surveys conducted make use of the common domain adaptation datasets, such as Office-31 (Amazon, Webcam, Digital) [21], Digits (MNIST, SVHN, MNIST-M) [6,22,23], and Syn2Real [24,25]. However, to our knowledge there is no DA study based on aerial datasets. Aerial imagery is important to the remote sensing community and presents unique challenges due to changes in rotation, resolution, illumination, and noise depending on the sensor characteristics. Moreover, models trained on ground based imagery do not generalize to aerial imagery due to some key differences. The viewpoints in aerial images are different than those in ground based images, as a the field of view of aerial cameras is larger and covers greater distance. This often means that the objects in aerial images consist of fewer pixels and are more difficult to describe. Other challenging conditions in aerial images include lower between-class variation, weather related disturbances such as cloud cover, and greater variations in the orientation of objects with respect to the background while still convey the same contextual information. These attributes make aerial datasets more challenging for generalization across domains and motivate the need for domain adaptation. In our benchmarking study, we focus on creating suitable aerial datasets for domain adaptation and report the results from seven different unsupervised domain adaptation methods on these datasets.

The main contributions of this paper are as follows:

1. We present the first benchmarking study of domain adaptation on aerial datasets.
2. We construct six different aerial datasets for domain adaptation by carefully selecting the common classes and balancing the datasets.
3. We consider seven models for unsupervised domain adaptation and report their performance on the aerial datasets.

The remainder of this work is organized as follows. Section 2 discusses the notation and categories of domain adaptation. Section 3 provides an explanation for each of the algorithms used for the benchmarking study. Section 4 describes the datasets and the process used to make the aerial domain adaptation datasets used in this study. Section 5 provides the comprehensive results from the study and confusion matrices and t-SNE [26] visualizations of the data before and after domain adaptation for each of the methods. Finally, Section 6 presents the final remarks and conclusions based on our evaluation.

## 2. Background

In unsupervised domain adaptation, the source domain where training takes place is described as $D_{s}={\left\{\left(x_{i}^{s},y_{i}^{s}\right)\right\}}_{i=1}^{n_{s}}$, where $n_{s}$ is the number of labeled samples, and the target domain, where testing takes place, is $D_{t}={\left\{\left(x_{j}^{t}\right)\right\}}_{j=1}^{n_{t}}$, where $n_{t}$ is the number of unlabeled samples. The number of classes is *K* in both source and target domains, and $C^{s}$ is the source classifier while $C^{t}$ is the target classifier. The feature extractor is described by F and the discriminator by D (when needed). Transfer learning (TL) was motivated by the insufficient training data problem, where the collection and labeling of data in some domains is expensive to do on a large scale. Here, the domains can be from the same modality [27] or different modalities [28,29]. TL refers to a broad category of algorithms, where knowledge transfer takes place, of which domain adaptation is a specific type.

Most DA methods can be categorized into divergence based and adversarial-based methods [10]. The divergence based DA works by minimizing the domain shift between the source and target distributions to obtain a domain invariant feature representation [10]. The classifier can then perform comparably on both domains. The commonly used methods for comparing the distribution shift and the works that use them are: maximum mean discrepancy (MMD) [4,30,31,32,33], Kullback-Leibler (KL) divergence [34], correlation alignment (CORAL) [35,36] and H divergence. Class labels can also be used to transfer knowledge between different domains [37,38,39,40], and if target labels are not available, pseudo labels are used [30,33,41]. Models that adjust the architecture of the model, such as adaptive batch normalization (ABN) [42,43,44], domain-guided dropout [45], etc., are also divergence based DA methods.

In the adversarial-based DA, a domain discriminator is used to encourage domain confusion by using an adversarial objective. This objective minimizes the distance between the empirical source and target mapping distributions. This is further categorized based on the use of generative versus nongenerative models. The generative model makes use of generative adversarial networks (GANs) to create synthetic target data, which is in turn used to train the target model [46,47,48]. In the nongenerative approach, the feature extractor learns a discriminative representation using the source domain labels and a domain-confusion loss. The domain-confusion loss is added with the classification loss and tries to match the source and target distributions to confuse the higher layers [6,37,49,50,51]. DANN [6] is a popular example of adversarial-based DA.

**Figure 1:**
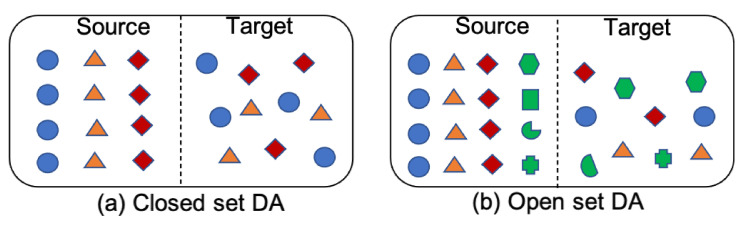
(**a**) Closed set DA has same classes in source and target domains. (**b**) Both source and target domains in open set DA contain contain data that do not belong to classes of interest. Unknown samples are represented in green. Further, target domain can contain data from classes not related to classes in source domain.

In most cases of TL and DA, the source and target domains have the same classes, and this is called closed set DA. When the classes in the source and target are not identical, the problem is called open set DA [52] and is more challenging. Figure 1 illustrates the difference between open set and closed set DA. The work presented here considers closed set unsupervised DA methods for classification.

All the DA algorithms that we have used for this benchmarking study were the top performing models with source code publicly available from each of the DA categories.

## 3. Domain Adaptation Algorithms

This section offers a description of each DA method used in our study. We begin with Domain Symmetric Networks (SymNets) [53], Robust Spherical Domain Adaptation (RSDA) [54], Conditional Adversarial Domain Adaptation with Gradually Vanishing Bridge as well as Gradually Vanishing Bridge [55], the unsupervised form of Universal Domain Adaptation [56], the source free method Source Hypothesis Transfer (SHOT) [57], and finally the Structurally Regularized Deep Clustering [58].

### 3.1. Domain-Symmetric Networks for Adversarial Domain Adaptation

The Domain Symmetric Network (SymNets) [53] utilizes a symmetric design of the source and target classifiers for adversarial unsupervised domain adaptation. SymNets make use of a novel adversarial learning method that includes a category-level and domain-level confusion loss that can enhance the learning of features to be domain-invariant for the various classes. The proposed cross-domain confusion scheme makes the target classifier symmetric to the source classifier in terms of predicting the classes. The domain-level confusion scheme used for the domain adversarial training makes use of the convolutional layers in the network as a feature extractor *G*, and the fully connected (FC) layers as the task classifier *C*. The domain discriminator *D*, which is symmetric to *C* is added on top of *G* to distinguish between the features of the samples from the two domains.

**Figure 2:**
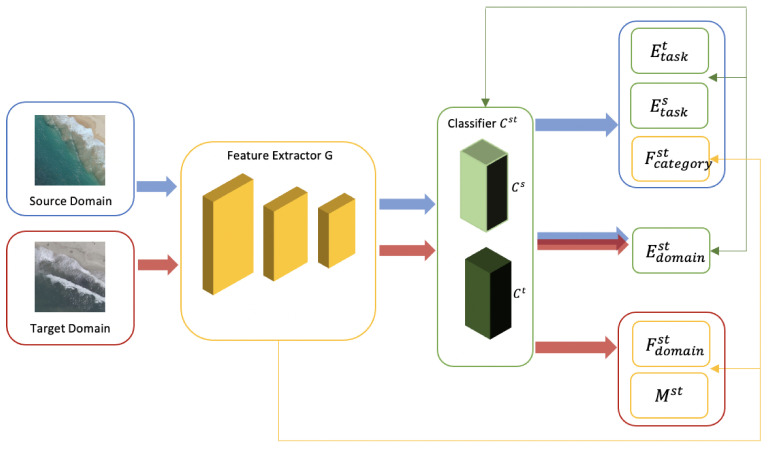
Architecture of SymNets [53]. Blue and red arrows indicate source and target domains and losses corresponding to them respectively. Yellow refers to feature extractor and corresponding losses and green represents classifiers and their losses.

The architecture of SymNets is described in Figure 2. In unsupervised domain adaptation, the source domain is described as $D_{s}={\left\{\left(x_{i}^{s},y_{i}^{s}\right)\right\}}_{i=1}^{n_{s}}$ containing $n_{s}$ labeled samples $x_{i}^{s}$ with labels $y_{i}^{s}$, and the target domain is $D_{t}={\left\{\left(x_{j}^{t}\right)\right\}}_{j=1}^{n_{t}}$ containing $n_{t}$ unlabeled samples $x_{j}^{t}$.

The SymNets design consists of two parallel task classifiers for *K* number of classes, $C^{s}$ and $C^{t}$, which are based on a single Fully Connected (FC) layer followed by softmax operations. The source task classifier $C^{s}$ is trained using the cross-entropy loss over the labeled source samples,

(1) $$\min_{C^{s}}E_{task}^{s}\left(G,C^{s}\right)=-\frac{1}{n_{s}}\sum_{i=1}^{n_{s}}log\left(p_{y_{i}^{s}}^{s}\left(x_{i}^{s}\right)\right),$$

where p(x)∈[0,1] is the probability of the sample belonging to a class after softmax. Since the target samples $x^{t}$ are unlabeled, the idea is to leverage the labeled source samples to train the target domain classifier $C^{t}$ using the cross-entropy loss (E):

(2) $$\min_{C^{t}}E_{task}^{t}\left(G,C^{t}\right)=-\frac{1}{n_{s}}\sum_{i=1}^{n_{s}}log\left(p_{y_{i}^{s}}^{t}\left(x_{i}^{s}\right)\right).$$

To make $C^{s}$ and $C^{t}$ distinguishable, domain discrimination training $C^{st}$ is done by making use of the two-way cross-entropy loss:

(3) $$\min_{C^{st}}E_{domain}^{st}\left(G,C^{st}\right)=-\frac{1}{n_{t}}\sum_{j=1}^{n_{t}}log\left(\sum_{k=1}^{K}p_{k+K}^{st}\left(x_{j}^{t}\right)\right)-\frac{1}{n_{s}}\sum_{i=1}^{n_{s}}log\left(\sum_{k=1}^{K}p_{k}^{st}\left(x_{i}^{s}\right)\right),$$

where $\sum_{k=1}^{K}p_{k}^{st}\left(x\right)$ and $\sum_{k=1}^{K}p_{k+K}^{st}\left(x\right)$ are the probabilities of classifying an input sample *x* as belonging to the source and target domains respectively.

The category-level confusion loss makes use of the labeled source samples and the feature extractor *G* is learned by the following objective:

(4) $$\min_{G}F_{category}^{st}\left(G,C^{st}\right)=-\frac{1}{2n_{s}}\sum_{i=1}^{n_{s}}log\left(p_{y_{i}^{s}+K}^{st}\left(x_{i}^{s}\right)\right)-\frac{1}{2n_{s}}\sum_{i=1}^{n_{s}}log\left(p_{y_{i}^{s}}^{st}\left(x_{i}^{s}\right)\right).$$

For the domain-level confusion loss, the unlabeled target samples are used as the individual class label is not required for domain-level confusion. For the target sample, the feature extractor *G* is learned by the following objective,

(5) $$\min_{G}F_{domain}^{st}\left(G,C^{st}\right)=-\frac{1}{2n_{t}}\sum_{j=1}^{n_{t}}log\left(\sum_{k=1}^{K}p_{k+K}^{st}\left(x_{j}^{t}\right)\right)-\frac{1}{2n_{t}}\sum_{j=1}^{n_{t}}log\left(\sum_{k=1}^{K}p_{k}^{st}\left(x_{j}^{t}\right)\right)$$

The entropy minimization principle [59] is used in SymNets to enhance the discrimination among task categories by summing over the probabilities at each pair of category-corresponding neurons in $C^{st}$,

(6) $$\min_{G}M^{st}\left(G,C^{st}\right)=-\frac{1}{n_{t}}\sum_{j=1}^{n_{t}}\sum_{k=1}^{K}q_{k}^{st}\left(x_{j}^{t}\right)log\left(q_{k}^{st}\left(x_{j}^{t}\right)\right),$$

where $q_{k}^{st}=p_{k}^{st}\left(x_{j}^{t}\right)+p_{k+K}^{st}\left(x_{j}^{t}\right)$, k∈[1,…,K].

The overall training objective is obtained by combining Equations (1)–(6) as,

(7) $$\begin{array}{c} \min_{C^{s},C^{t},C^{st}}E_{task}^{s}\left(G,C^{s}\right)+E_{task}^{t}\left(G,C^{t}\right)+E_{domain}^{st}\left(G,C^{st}\right)+\min_{G}F_{category}^{st}\left(G,C^{st}\right)\\ +\lambda (\min_{G}F_{domain}^{st}\left(G,C^{st}\right)+\min_{G}M^{st}\left(G,C^{st}\right)), \end{array}$$

where λ∈[0,1] is a trade-off parameter to suppress noisy signals from $F_{domain}^{st}\left(G,C^{st}\right)$ and $M^{st}\left(G,C^{st}\right)$ during the early stages of training.

### 3.2. Spherical Space Domain Adaptation with Robust Pseudo-Label Loss

**Figure 3:**
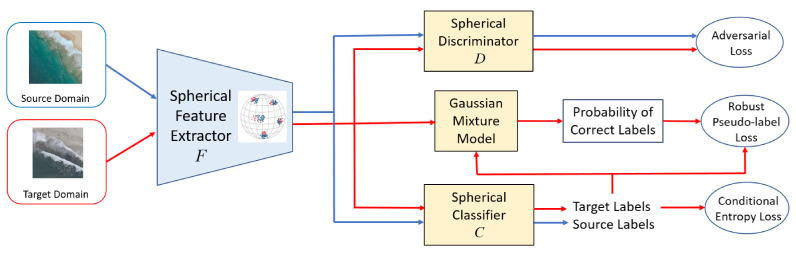
Architecture of RSDA [54]. Blue and red arrows represent computational flow of source and target domain samples respectively. *F* is a feature extractor which is a CNN that extracts features and embeds them onto a hypersphere. Spherical classifier predicts class labels and domain discriminator predicts domain labels. Posterior probability of correct labels is obtained by feeding the target pseudo-labels and target features into a Gaussian mixture model. Posterior probabilities then weight pseudo-label loss for robustness.

Robust Spherical Domain Adaptation (RSDA) [54] proposes a novel adversarial domain adaptation approach by leveraging the spherical space and defining a spherical neural network. A robust pseudo-label loss is defined to make effective use of the pseudo-labels. This loss weighs the importance of the estimated labels on target data by the posterior probability of the correct label that is modeled by a Gaussian-uniform mixture model in spherical space. The spherical features ($L_{2}$ normalized) were shown to improve performance in recognition and domain adaptation [60,61,62,63,64]. RSDA extends this idea by defining all the operations in spherical feature space to leverage the advantages of the spherical space structure. The architecture of RSDA is described in Figure 3.

The source domain has a labeled dataset, ${\left\{x_{i}^{s},y_{i}^{s}\right\}}_{i=1}^{N_{s}}$ and the target domain is unlabeled, ${\left\{x_{j}^{t}\right\}}_{j=1}^{N_{t}}$. The goal of RSDA is to transfer the knowledge obtained from the labeled source data classifier to get target labels. The feature extractor *F* goes through adversarial training with a domain discriminator *D* such that *F* is able to distinguish between the source and target domains. A CNN such as ResNet [65] is used as the feature extractor *F* which is mapped onto a sphere. The spherical feature space also has a classifier (*C*) and discriminator (*D*). The spherical neural network consists of spherical perceptron and spherical logistic regression layers. The robust pseudo-label loss defined in spherical space makes use of the pseudo-labels of the target domain and the Gaussian mixture model.

The spherical adversarial training loss is defined as,

(8) $$L=L_{bas}\left(F,C,D\right)+L_{rob}\left(F,C,\phi \right)+\gamma L_{ent}\left(F\right),$$

and takes into account the basic loss, robust pseudo-label loss, and conditional cross entropy loss, which are all defined in the spherical feature space. $L_{bas}\left(F,C,D\right)$ is the basic loss which is used to learn the classifier in the source domain and align features across domains. The basic adversarial domain adaptation loss takes either DANN [6] or MSTN [66] as the baseline and $L_{bas}$ is the spherical version of the loss. The cross-entropy loss, $L_{ent}\left(F\right)$ is used to reduce prediction uncertainty and is defined as,

(9) $$L_{ent}\left(F\right)=\frac{1}{N_{t}}\sum_{j=1}^{N_{t}}H\left(C\left(F\left(x_{j}^{t}\right)\right)\right),$$

where *H* is the entropy of the distribution. To define the form of the robust pseudo-label loss, $L_{rob}\left(F,C,\phi \right)$, the pseudo label is ${\tilde{y}}_{j}^{t}=argmax_{k}{\left[C\left(F\left(x_{i}^{s}\right)\right)\right]}_{k}$ for the *k*th element. A random variable $z_{j}\in \left\{0,1\right\}$ is used to figure out if the data are correctly labeled (1) or wrongly labeled (0). If the probability of correct labeling is $P_{\phi }\left(z_{j}=1|x_{j}^{t},{\tilde{y}}_{j}^{t}\right)$ with parameter ϕ, the robust loss is,

(10) $$L_{rob}\left(F,C,\phi \right)=\frac{1}{N_{0}}\sum_{j=1}^{N_{t}}w_{\phi }\left(x_{j}^{t}\right)J\left(C\left(F\left(x_{j}^{t}\right)\right),{\tilde{y}}_{j}^{t}\right),$$

where, $N_{0}=\sum_{j=1}^{N_{t}}w_{\phi }\left(x_{j}^{t}\right)$, and J(·,·) is the mean absolute error (MAE) [67]. $w_{\phi }\left(x_{j}^{t}\right)$ is defined based on the posterior probability of correct labeling,

$$w_{\phi }\left(x_{j}^{t}\right)=\left\{\begin{array}{cc} \gamma_{j}, & \mathrm{if}\;\gamma_{j}\geq 0.5,\\ 0, & \mathrm{otherwise}, \end{array}\right.$$

where $\gamma_{j}=P_{\phi }\left(z_{j}=1|x_{j}^{t},{\tilde{y}}_{j}^{t}\right)$.

The probability $P_{\phi }\left(z_{j}=1|x_{j}^{t},{\tilde{y}}_{j}^{t}\right)$ is learned using the Gaussian mixture model in spherical feature space.

The spherical neural network (SNN) is an extension of MLP from Euclidean to spherical space. The features on the spherical space are obtained by normalizing the feature vectors, $f=r\frac{F(x)}{\left||F(x)|\right|}$. The classifier is constructed by stacking a few spherical perceptron (SP) layers and a final spherical logistic regression (SLR) layer. The SP layer consists of a linear transform and an activation function.

The spherical linear transform (g) consists of first projecting the features from the former spherical surface onto the tangent plane, then a linear transformation to transform the projected features onto the tangent plane of the later spherical surface, and then back from the tangent space onto the later spherical surface. The nonlinear activation function in spherical space is defined as,

(11) $$SReLU\left(x\right)=r\frac{ReLU(x)}{\left||ReLU(x)|\right|}.$$

The SP layer is then,

(12) $$f_{out}=SReLU\left(g\left(f_{in}\right)\right).$$

The SLR layer is described similar to the Euclidean logistic regression as,

(13) $$p\left(y=k|z\right)\propto exp\left(w_{k}^{T}z+b_{k}\right),k=1,2,\ldots ,K,$$

where, $w_{k}^{T}z+b_{k}=0$ is the classification boundary on the sphere.

### 3.3. Gradually Vanishing Bridge for Adversarial Domain Adaptation

Gradually Vanishing Bridge for Adversarial Domain Adaptation [55] proposes a method to perform unsupervised adversarial domain adaptation that utilizes a vanishing bridge mechanism on the generator and the discriminator used in the network. The bridge is a measurement which models the difference between the existing and ideal representation of the domains. On the generator, the bridge reduces the overall transfer difficulty and reduces the influence of residual domain specific characteristics. It connects either the source or the target to an intermediate domain to enable domain alignment. On the discriminator, the bridge enhances the discriminating ability and balances the adversarial training process. During the training process, the range of the bridge is reduced gradually to reduce the influence of the domain characteristics of the bridge output which in turn reduces the discrepancy between the source and target. After the adaptation is done, more points from both the domains are covered in the intermediate domain. The points that are still outside of the intermediate domain are taken as hard examples. Applying the bridge to both the generator and discriminator is denoted as GVB-GD and it ensures that the two-player minmax game is balanced. The adversarial training process also ensures that the distribution of the intermediate representation is similar across both the domains.

The gradually vanishing bridge framework is an end-to-end network. The bridge layer on the generator outputs the domain specific properties, called γ. The intermediate representation $r_{i}$ is found by subtracting γ from the classifier response $c_{i}$. This representation is minimized by the classification loss.

(14) $$\begin{array}{c} r_{i}=c_{i}-\gamma_{i} \end{array}$$

The bridge layer associated with the discriminator represents the distance between the current discriminator function and the ideal decision boundary to be achieved. The discriminator also receives additional discriminative power from the bridge layer. The overall objective of the network is given by the equation below which shows that the network is trained by minimizing classification loss, adversarial transfer loss, and the reconstruction loss for the generator and maximizing the adversarial transfer loss for the discriminator.

(15) $$\begin{array}{c} \min_{G_{*}}\left(L_{cls}+L_{trans}^{adv}+L_{ext}\right)\\ \max_{D_{*}}L_{trans}^{adv} \end{array}$$

where $L_{cls}$ is the classification loss, $L_{trans}^{adv}$ is the adversarial transfer loss and $L_{ext}$ is the reconstruction loss.

### 3.4. Conditional Adversarial Domain Adaptation with Gradually Vanishing Bridge

Gradually Vanishing Bridge [55] can be applied to other unsupervised domain adaptation methods like Conditional Adversarial Domain Adaptation (CDAN) [68]. CDAN uses conditioning strategies such as multilinear conditioning that captures the cross variance between feature representations and classifier predictions to improve the discrimination process. In the model CDAN-GD, the gradually vanishing bridge is applied on the generator and discriminator. The major contribution of CDAN is the domain discriminator, which is trained on the cross-covariance of the domain specific feature representations and predictions made by the classifier. This discriminator is also trained on the ambiguity of the classifier which helps the discriminator prioritize the easier samples. Back propagation is then used to solve the system in linear time.

This method improves adversarial domain adaptation methods by addressing two shortcomings. First, when the joint distributions of features and classes across both domains are not identical, adapting only the feature representation will not be enough as deep representations usually transition from general to specific as training progresses in deep networks. The second shortcoming occurs when the feature distribution is multi-modal, which often occurs in real-life scenarios. In this case, the adversarial network performs poorly. These challenges are overcome by training the generator and discriminator on information related to the domains such as its respective labels or similar modality objects. By adapting the feature representation *f*, the classifier prediction *g* is able to capture multi-modal structures in the adversarial domain adaptation. Domain variations in both feature representation *f* and classifier prediction *g* can be represented at the same time via conditioning. Thus, CDAN is presented as a minimax optimization problem consisting of two error terms: E(G) from the source classifier *G*, which needs to be minimized for reduced source risk, and E(D,G), which is related to the source classifier *G* and the domain discriminator *D* across both domains. Here, E(D,G) will be minimized over the discriminator but maximized over the feature representation f=F(x) and classifier prediction g=G(x). The minimax game of conditional domain adversarial network (CDAN) is shown as,

(16) $$\begin{array}{c} \min_{G}E\left(G\right)-\lambda E\left(D,G\right)\\ \min_{D}E\left(D,G\right) \end{array}$$

where λ is a hyper-parameter between the two objectives to trade off source risk and domain adversary.

### 3.5. Universal Domain Adaptation with Universal Adaptation Network

Most domain adaptation techniques require a definite class-wise relationship between the source and target domain classes. Universal Adaptation Network (UAN) [56] is an interesting modification of DANN [6] where the network does not need to know any specific class-wise relationship between the source and target domains. UAN can identify the target samples that do not belong to any of the classes of the source domain and categorize them as unknown samples, while classifying other target samples into one of the source domain classes.

In UAN, during training, a source domain $D_{s}=\left(x_{i}^{s},y_{i}^{s}\right)$ with $n_{s}$ annotated samples of $C_{s}$ classes collected from a distribution *p* and a target domain $D_{t}=\left(x_{i}^{t}\right)$ with $n_{t}$ non-annotated samples of $C_{t}$ collected from a distribution *q* are given. The common label set shared by source and target domains is denoted by $C=C_{S}⋂C_{t}$. The label sets or classes specific to the source and target domains are denoted by $\bar{C_{s}}=C_{s}$\*C* and $\bar{C_{t}}=C_{t}$\*C*, respectively. The target data are totally nonannotated, and the target label set is defined only for the purpose of explanation of the setting. Commonness between the source and target domains is defined as the Jaccard distance of the label sets $\xi =\frac{|C_{s}⋂C_{t}|}{|C_{s}⋃C_{t}|}$. In our case of aerial datasets under consideration, keeping in line with all other methods evaluated, we selected a closed setting with ξ=1.

The Universal Adaptation Network consists of a feature extractor *F*, classifier *G*, adversarial domain discriminator *D* and a nonadversarial domain discriminator $D^{\prime }$. Inputs *x* from both domains are processed through the feature extractor *F* to get features z=F(x), which are then fed into the classifier *G* to get the probability $\hat{y}=G\left(z\right)$ over the source classes. In addition to domain gap, in a universal setting there is also category gap between source and target domains. To mitigate this discrepancy, UAN introduces the non-adversarial discriminator $D^{\prime }$ that learns to calculate the domain similarity parameter $\hat{d^{\prime }}$. It is, in turn, used to compute the source transferability criterion for training the network with the adversarial discriminator *D*. Source similarity weights $w^{s}$ and $w^{t}$ of the source and target domains, respectively, are calculated following the equation below, under the assumption that entropy expresses the uncertainty in predictions and more confident predictions produce lower entropy.

(17) $$\begin{array}{c} w^{s}\left(x\right)=\frac{H(\hat{y})}{log|C_{s}|}-\hat{d^{\prime }}\left(x\right)\\ w^{t}\left(x\right)=\hat{d^{\prime }}\left(x\right)-\frac{H(\hat{y})}{log|C_{s}|} \end{array}$$

Here, $H(\hat{y})$ is the entropy of the sample prediction and $\hat{d^{\prime }}\left(x\right)$ is the output of non-adversarial discriminator $D^{\prime }$. Weights $w^{s}$ and $w^{t}$ confine domain alignment via adversarial training within the shared label set between the two domains. The error functions can therefore be written as follows.

(18) $$\begin{array}{c} E_{G}=E_{(x,y)∼p}L\left(y,G\left(F\left(x\right)\right)\right)\\ E_{D^{\prime }}=-E_{x∼p}logD^{\prime }\left(F\left(x\right)\right)-E_{x∼q}log\left(1-D^{\prime }\left(F\left(x\right)\right)\right)\\ E_{D}=-E_{x∼p}w_{s}\left(x\right)logD\left(F\left(x\right)\right)-E_{x∼q}w_{t}\left(x\right)log\left(1-D\left(F\left(x\right)\right)\right) \end{array}$$

where *L* is the cross-entropy loss. Therefore, the objective functions are the following.

(19) $$\begin{array}{c} \max_{D}\min_{F,G}E_{G}-\lambda E_{D}\\ \min_{D^{\prime }}E_{D^{\prime }} \end{array}$$

where λ is a hyperparamter for trade-off between domain transferability and discriminability. In the test phase, the adversarial discriminator is removed while the nonadversarial discriminator, along with the classifier produce the source similarity weights and depending on a source similarity threshold, the samples correspond to one of the source classes or as an unknown.

### 3.6. Source Hypothesis Transfer

**Figure 4:**
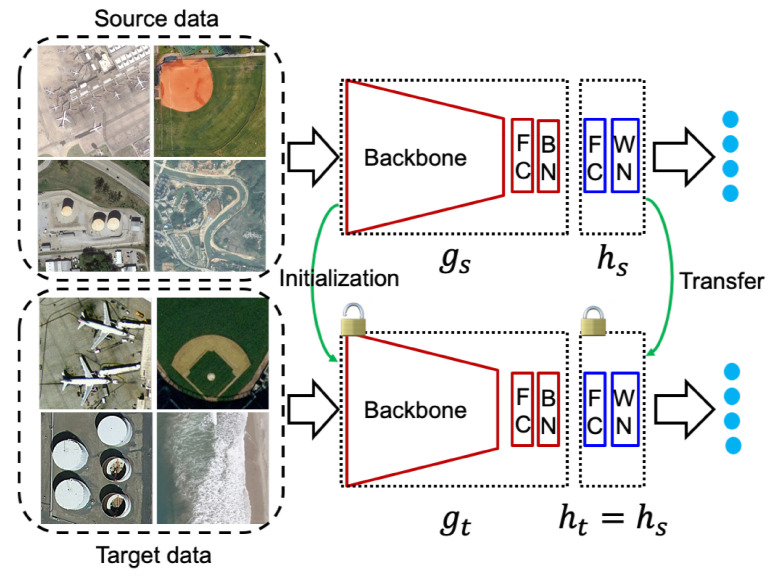
Network architecture of Source Hypothesis Transfer (SHOT) [57].

Source Hypothesis Transfer (SHOT) proposes a discrepancy based domain adaptation technique using hypothesis transfer from source domain $D_{s}$ to the target domain $D_{t}$. For vanilla unsupervised domain adaptation task, $n_{s}$ labeled samples ${\left\{x_{s}^{i},y_{s}^{i}\right\}}_{i=1}^{n_{s}}$ are given where $x_{s}^{i}\in X_{s}$, $y_{s}^{i}\in Y_{s}$ with $n_{t}$ target samples ${\left\{x_{t}^{i}\right\}}_{i=1}^{n_{t}}$ where $x_{t}^{i}\in X_{t}$. For vanilla unsupervised DA, the goal is to find ${\left\{y_{t}^{i}\right\}}_{i=1}^{n_{t}}$ where $y_{t}^{i}\in Y_{t}$ for the target samples by learning a mapping $f_{t}:X_{t}\to Y_{t}$. SHOT proposed a domain adaptation technique without the source data during the adaptation procedure. To achieve that, first a network is trained with a source data to learn the mapping $f_{s}:X_{s}\to Y_{s}$. During the adaptation procedure with the target data, the source trained model is initialized and the parameters for the classifier part of the model is kept frozen and only the backbone part of the network is trained with the information maximization (IM loss). The overall architecture of the SHOT method is shown in Figure 4.

During training the network on the source domain $D_{s}$, the source feature extractor $g_{s}$ and the source hypothesis $h_{s}$ are both trained. Here, $g_{s}$ encodes the input image to a *d* dimension feature, i.e., $g_{s}:X_{s}\to R^{d}$ and the hypothesis module takes the embedding feature and output *k* dimensional logits, i.e., $h_{s}:R^{d}\to R^{k}$, where the total number of classes is *k*. During the domain adaptation procedure on the target domain $D_{t}$, the target feature extractor $g_{t}$ is initialized with the source trained backbone and learned during the domain adaptation procedure. The source hypothesis $h_{s}$ is transferred to the target hypothesis $h_{t}$ and keeps unchanged throughout the network training procedure.

The loss function for the source training is categorical cross-entropy loss with label smoothing. The label smoothing component helps to achieve evenly separated clustering in the embedding space. The overall source training loss can be written as follows.

(20) $$L_{src}\left(f_{s};X_{s},Y_{s}\right)=-E_{\left(x_{s},y_{s}\right)\in X_{s}\times Y_{s}}\sum_{k=1}^{K}q_{k}\log\delta_{k}\left(f_{s}\left(x_{s}\right)\right)$$

where, $\delta_{k}=\frac{exp(a_{k})}{\sum_{i}exp\left(a_{i}\right)}$, during training with the target data, as the source data are not made available, the distribution alignment based on features does not work. The authors argue that the ideal output probability for the target samples should be similar to one hot vectors, i.e., the model should be confident and diverse. For this purpose, the information maximization (IM) loss is introduced as the sum of the entropy loss $L_{ent}$ and KL-divergence loss $L_{div}$ and can be written as follows.

(21) $$\begin{array}{c} L_{ent}\left(f_{t};X_{t}\right)=-E_{x_{t}\in X_{t}}\sum_{k=1}^{K}\delta_{k}\left(f_{t}\left(x_{t}\right)\right)\log\delta_{k}\left(f_{t}\left(x_{t}\right)\right)\\ L_{div}\left(f_{t};X_{t}\right)=\sum_{k=1}^{K}{\hat{p}}_{k}\log{\hat{p}}_{k} \end{array}$$

where, $f_{t}\left(x\right)=h_{t}\left(g_{t}\left(x\right)\right)$ is the output logits of the network with *K*-dimensions and $\hat{p}=E_{x_{t}\in X_{t}}\left[\delta \left(f_{t}\left(x_{t}\right)\right)\right]$ is computed as the mean of the entire target domain. However, this does not ensure that similar classes match with each other between the source and the target domain. To alleviate this, the authors propose to use self supervised pseudo labeling motivated by deep clustering technique. They compute the class centroid for each of the target classes and compute the pseudo labels based on the cosine distances from each of the centroids. The overall loss function for the domain adaptation can be written as follows.

(22) $$\begin{array}{c} L_{ent}\left(g_{t}\right)=L_{ent}\left(f_{t};X_{t}\right)+L_{div}\left(f_{t};X_{t}\right)-\\ \beta E_{\left(x_{t},{\hat{y}}_{t}\right)\in X_{t}\times {\hat{Y}}_{t}}\sum_{k=1}^{K}1_{\left[k={\hat{y}}_{t}\right]}\log\delta_{k}\left(f_{s}\left(x_{s}\right)\right) \end{array}$$

where ${\hat{y}}_{t}\in {\hat{Y}}_{t}$ is the estimated pseudo labels. Recently Taufique et al. [69] proposed a variant of SHOT utilizing HRNet [70] backbone that further improved the performance of SHOT.

### 3.7. Unsupervised Domain Adaptation via Structurally Regularized Deep Clustering

Structurally Regularized Deep Clustering (SRDC) [58] is a discrepancy based unsupervised domain adaptation method which minimizes the risk of damaging the intrinsic discrimination of target data. This potential damaging of the target domain usually occurs when the domain aligned features are explicitly learned. To avoid this, a source regularized, deep discriminative clustering method is used to directly show the existing discrimination within the target data. The method is motivated by the assumption that structural similarity exists between the two domains. This method uses a framework of deep network based discriminative clustering that minimizes the KL divergence between the predictive label distribution of the network and an introduced auxiliary one, by replacing the auxiliary distribution with the ground truth labels of the source data. Thus, it implements the structural source regularization with joint network training. The target discrimination process is also enhanced by clustering of the intermediate network features. The structural regularization is also enhanced by selecting less divergent source examples.

The joint network method consists of an embedding function and a classifier function. The assumptions for the method are that the classes used are discriminative within a domain but the same classes in different domains are geometrically close. After getting the softmax predictions of the target data, the network minimizes the KL divergence between the softmax outputs of the target samples and the auxiliary samples. The method then alternates between updating the auxiliary samples and using these updated samples to train the network to update the network parameters to optimize deep clustering. The equation below shows the objective of deep clustering that is to be achieved. The first term calculates the KL divergence where $P^{t}$ is the softmax output of the target samples and $Q^{t}$ is the softmax output of the auxiliary samples. The second term in the equation is used to balance the cluster assignment. This term also helps in maintaining the cluster size balance by performing entropy maximization of the labels on the target domain.

(23) $$\begin{array}{c} \min_{Q^{t},\theta ,\nu }L^{t}=KL(Q^{t}\left|\right|P^{t})+\sum_{k=1}^{K}Q_{k}^{t}logQ_{k}^{t},\\ whereQ_{k}^{t}=\frac{1}{n_{t}}\sum_{i=1}^{n^{t}}q_{i}^{t},k \end{array}$$

The above objective is optimized by alternating between the auxiliary distribution step and the network update step. Since both the domains share the same label space, joint training pushes instances from the same classes into the same regions, thus achieving feature alignment between the two domains. The clusters are also enhanced by selecting soft source samples, and this is done by reassigning the weights to the source after every epoch, based on their similarity to the target samples.

## 4. Aerial Datasets

This section describes the aerial datasets used in this benchmarking study. Since there are no aerial datasets dedicated to domain adaptation, we considered publicly available aerial datasets for classification and utilized their shared classes for unsupervised domain adaptation.

### 4.1. AID

The Aerial Image Dataset (AID) [71] is a dataset developed for the task of aerial scene classification by procuring images from Google Earth and contains 30 classes of aerial or satellite imagery: beach, bridge, center, airport, bare land, baseball field, church, commercial, dense residential, forest, industrial, meadow, desert, farmland, medium residential, mountain, park, port, railway station, resort, parking, playground, pond, river, school, sparse residential, storage tanks, viaduct square and stadium. The annotations were made by experts in remote sensing image interpretation. The images in the dataset are termed as multisource images as the Google Earth images are collected from varying remote imaging sensors. The images were selected from many countries some of which are, the United States, England, Italy, China, Japan, France, and Germany. The data are also considered diverse as the images were captured under different imaging conditions such as at different times during the day and at various seasons throughout the year. There are a total of 10,000 aerial images of size 600 × 600 pixels. The images are obtained at multiple ground sampling distances (GSDs) ranging from 8 m to 0.5 m. The classes selected from AID for our experiments are airport, parking, storage tank, beach, forest, river, baseball field, medium residential, and sparse residential.

### 4.2. UCM

The UC Merced Land Use Dataset (UCM) [72] is a publicly available image dataset of overhead land images meant for research purposes. It consists of 21 classes and has 100 images per class measuring 256 × 256 pixels. The pixel resolution of the dataset is 1 foot/0.3 m per pixel in the RGB color space. The dataset consists of these classes: beach, buildings, chaparral, agricultural, airplane, baseball diamond, dense residential, forest, freeway, golf course, mobile home park, overpass, parking lot, harbor, intersection, medium residential, river, runway, sparse residential, tennis courts and storage tanks. The images were downloaded from the United States Geological Survey (USGS) National Map from different urban US regions. The images selected contain a wide variety of spatial patterns, textures and colors making it ideal for scene classification. The classes selected from UCM for our experiments are airplane, parking lot, storage tank, beach, forest, river, baseball diamond, medium residential, and sparse residential.

### 4.3. NWPU

The NWPU-RESISC45 [73] dataset was created by Northwestern Polytechnical University (NWPU) for REmote Sensing Image Scene Classification (RESISC). It is known for having high diversity within each class while also maintaining similarity amongst the classes. The dataset was collected from Google Earth. NWPU has a total of 31,500 high-resolution remote sensing images which are divided into 45 scene classes. The classes are as follows: circular farmland, cloud, commercial area, dense residential, desert, forest, freeway, golf course, ground track field, airplane, airport, baseball diamond, basketball court, beach, bridge, chaparral, church, harbor, industrial area, intersection, island, lake, meadow, medium residential, mobile home park, roundabout, runway, sea, ice, ship, snowberg, sparse residential, stadium, storage tank, mountain, overpass, palace, parking lot, railway, railway station, rectangular farmland, river, tennis court, terrace, thermal power station, and wetland. Each class has 700 RGB images each of size 256 × 256 pixels. The spatial resolution is from around 30 m to 0.2 m per pixel. The classes selected from NWPU for our experiments are railway station, parking lot, bridge, runway, storage tank, and airplane.

### 4.4. CLRS

The Continual Learning Benchmark for Remote Sensing (CLRS) [74] dataset was designed for remote sensing image scene classification and for continual/lifelong learning. The authors created a criterion for three continual learning scenarios and have divided the dataset into those three categories. The CLRS dataset has 25 classes and a total of 15,000 images. The remote sensing images were procured from Google Earth, Bing Map, Google Map and Tianditu which all possess different remote imaging sensors, so the images are multi-source. Each class contains 600 images of size 256 × 256 pixels. The resolution of the data ranges from 0.26 m to 8.85 m. The 25 scene classes are highway, industrial, meadow, airport, bare-land, beach, mountain, overpass, park, parking, playground, commercial, desert, farmland, port, railway, railway-station, residential, river, runway, forest, golf-course, stadium, and storage-tank. The classes of interest from CLRS for our work are railway station, parking, bridge, runway, storage tank, and airport.

### 4.5. xView

This dataset was created as part of the xView [75] 2018 Detection Challenge. It contains around 1 million object samples divided across 60 classes with the option of using either 3-band or 8-band imagery. The images have a resolution of 0.3 m/pixel. This is an imbalanced dataset as there are some classes with many instances and some classes with only a few instances. The images are captured using the WorldView-3 satellite at 0.3 m ground sample distance. The objects within each image in this dataset vary in size from 3 m to greater than 3000 m. Each image in this dataset is of a very high resolution and often there were multiple objects from different classes within an image. This makes it difficult to perform classification accurately. To overcome this, each image was cropped around the bounding boxes so that only 1 object is in each image from a single class. The original images range from 2500 × 2500 to 4000 × 4000 pixels. The cropped image sizes range from 10 × 10 to 987 × 987. More preprocessing was done on this dataset to achieve optimum results for our experiments. The images which were smaller than 30 × 30 pixels were discarded. The number of images per class was restricted to 5000 and for classes not meeting this requirement, data augmentation was performed. The data augmentation was in the form of flipping the image horizontally and vertically. The final dataset contained the classes small vehicle, large vehicle, storage tank, plane, and ship. The classes which were augmented are plane, ship and storage tank.

### 4.6. DOTA

The Dataset for Object deTection in Aerial images (DOTA) [76] dataset is a benchmark dataset created for performing object detection in aerial images. The images in this dataset are mainly gathered from Google Earth and satellite JL-1 and satellite GF-2, which belong to the Chine Centre for Resources Satellite Data and Application. A total of 2086 images were captured using these satellites from different areas of the world. The images are in a range of around 800 × 800 pixels to 6000 × 6000 pixels. The object categories in this dataset are ground track field, harbor, bridge, large vehicle, small vehicle, helicopter, roundabout, soccer ball field, swimming pool, plane, ship, storage tank, baseball diamond, tennis court and basketball court. Similar to xView, each image in this dataset is of a very high resolution and often there were multiple objects from different classes within an image. This makes it difficult to perform classification accurately. To overcome this, each image was cropped around the bounding boxes so that only 1 object is in each image from a single class. The cropped image sizes range from 10 × 10 to 904 × 904. More preprocessing was done on this dataset to achieve optimum results for our experiments. The images which were smaller than 30 × 30 pixels were discarded. The number of images per class was restricted to 5000 and for classes not meeting this requirement, data augmentation was performed. The data augmentation was in the form of flipping the image horizontally and vertically. The final dataset contained the classes small vehicle, large vehicle, storage tank, plane, and ship. The class which was augmented is storage tank.

### 4.7. Aerial DA Datasets

**Figure 5:**
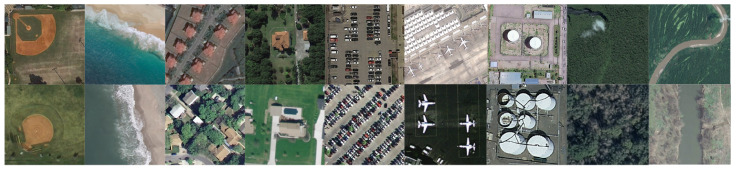
Sample images from shared classes between AID (**top row**) and UCM (**bottom row**). Classes are (from left to right) baseball field/baseball diamond, beach, medium residential, sparse residential, parking/parking lot, airport/airplane, storage tank, forest, and river.

To the best of our knowledge, there are no existing datasets designed for aerial domain adaptation. For closed-set DA, the classes between the source and target domain datasets are the same. Using the datasets described in this section, we create three aerial DA datasets for our benchmarking study. Each of these datasets are made by taking the common classes between two of the aerial datasets. There are differences in the image characteristics between the source and target domain based on their GSDs and the sensors that were used to collect the data. The first DA dataset is between AID and UCM, with 9 classes in common between them. The shared classes and number of samples for the AID-UCM DA dataset is in Table 1. Sample images from the shared classes are shown in Figure 5.

**Figure 6:**
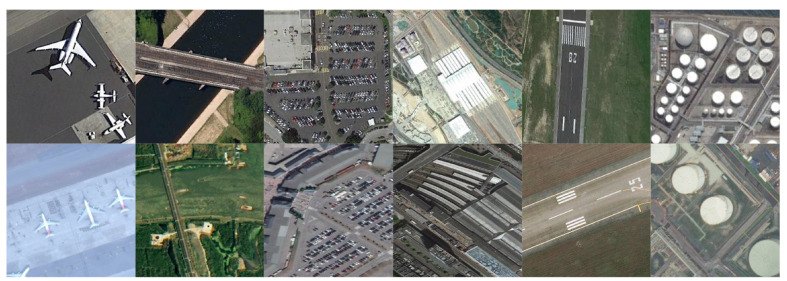
Sample images from shared classes between NWPU (**top row**) and CLRS (**bottom row**). Classes are (from left to right) airplane, bridge, parking, railway station, runway, and storage tank.

The next dataset is created by considering the common classes between NWPU and CLRS. The 6 shared classes and samples in each class for the NWPU-CLRS DA dataset is in Table 2. Sample images from the shared classes are shown in Figure 6.

**Figure 7:**
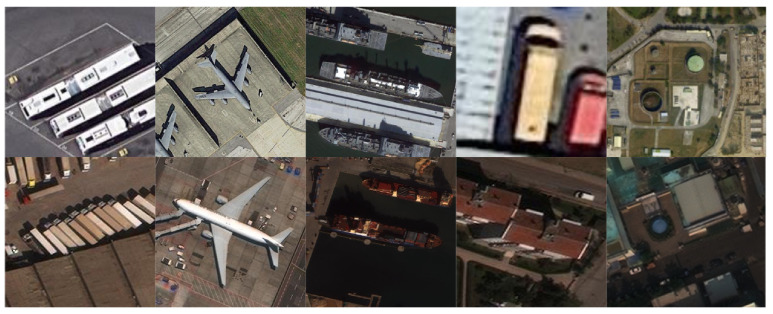
Sample images from shared classes between DOTA (**top row**) and xView (**bottom row**). Classes are (from left to right) large vehicle, plane, ship, small vehicle, and storage tank.

The third dataset is created by taking the common classes between DOTA and xView. The 5 shared classes and samples in each class for the DOTA-xView DA dataset is in Table 3. Sample images from the shared classes are shown in Figure 7.

## 5. Experiments

The code for the DA methods in our study was obtained from the URLs in Table 4. We considered the entire source domain for training and the entire target domain for adaptation. All the methods are unsupervised DA, so no label information from the target domain is used during adaptation. After adaptation, we consider the entire target domain for evaluation. The implementation for SymNets, RSDA, SRDC, CDAN-GD, and GVB-GD was done on a Linux workstation with an NVIDIA Titan V GPU with 12 GB memory. SHOT and UAN were implemented on a Linux workstation with an RTX 2080 Ti GPU with 12 GB memory. The parameter selection of the specific methods is described as follows.

**SymNets**: the backbone and implementation of SymNets follows the original described in [53]. The feature extractor *G* is a pretrained ResNet-50 [65] excluding the last FC layer. The feature extractor *G* is fine-tuned depending on the dataset and the classifier $C^{st}$ is trained from scratch using back-propagation. The learning rate of $C^{st}$ is 10 times that of *G*. The optimization used is SGD with a momentum of 0.9 and batch size of 128. The strategy described in [53] to update the learning rate (λ) is used. The learning rate is adjusted using $\eta_{p}=\frac{\eta_{0}}{{\left(1+\alpha p\right)}^{\beta }}$, where *p* is the progress of training epochs linearly changing from 0 to 1, $\eta_{0}=0.01$, α=10 and β=0.75. λ is gradually changed from 0 to 1 by $\lambda_{p}=\frac{2}{1+exp(-\gamma p)}-1$ and γ=10 for all the experiments.

**RSDA**: the backbone network architecture was kept the same as the original RSDA implementation in [54]. The training is done by alternatively optimizing the network parameters *F*, *C*, *D* and the parameters ϕ of the Gaussian mixture models while freezing one of the sets of parameters. The network is first trained with the basic loss from DANN [6] $L_{bas}$ to initialize *F*, *C*, and *D*. The following procedures are then run alternately:

1. Fix *F*, *C*, and *D* and estimate ϕ: The pseudo-labels ${\tilde{y}}_{j}^{t}$ are estimated by fixing *F*, *C*, and *D* and the distance of a sample from the spherical class center for a class ${\tilde{y}}_{j}^{t}$ is calculated using the cosine distance. Then, ϕ is estimated by the EM algorithm [59].
2. Optimizing *F*, *C*, and *D* and fixing ϕ: With the current pseudo-labels and parameter ϕ, *F*, *C*, and *D* are trained as a standard domain adaptation training by the progressive adversarial training strategy described in [6] with the objective function in Equation (8).

The feature extractor, *F*, is set to a ResNet-50 [65] pretrained on Image-Net excluding the last FC layer. Optimizing *F*, *C*, and *D* is done by SGD with a momentum of 0.9 and learning rates of *C* and *D* and 10 times that of *F*. Following the method in [6], the learning rate η and hyperparameter γ are estimated by $\eta =\frac{0.01}{{\left(1+\alpha p\right)}^{\beta }}$ and $\gamma =\frac{2}{1+exp(-\tau p)}-1$, where α=10, β=0.75, τ=10, and *p* is the optimizing progress that linearly changes from 0 to 1. The alternating iteration is performed 10 times and each step runs SGD for 5000 steps.

**CDAN-GD**: for these experiments, the backbone network is a ResNet-50 [65] which is pretrained on ImageNet, as done in the original paper. The new layers and classifier layers are trained through back propagation, where the classifier is trained from scratch with the learning rate set as 10 times that of the lower layers. As with GVB-GD the bridge layer is built on the generator and discriminator using fully connected layers. The (λ) is set to 1 and the (µ) is set to 1 to indicate the bridge on both networks. The CDAN is also implemented with entropy conditioning which prioritizes the discriminator on easy to transfer samples. For the training process, we employ mini-batch SGD with the momentum of 0.9 and a weight decay of 0.0005. The learning rate is taken as 0.001 for all the experiments with a batch size of 16.

**GVB-GD**: in these experiments, the backbone network is a ResNet-50 [65] which is pretrained on ImageNet, as done in the original paper. The bridge layer is built on the generator and discriminator using fully connected layers. In the adversarial training gradient reversal layer is applied to the network. In the early stages of the training procedure, a progressive training strategy is applied to suppress noisy signals from the discriminator. For the training process we employ mini-batch stochastic gradient descent (SGD) with the momentum set to 0.9 and a weight decay of 0.0005. The learning rate is taken as 0.001 for all the experiments with a batch size of 16. The (λ) is set to 1 and the (µ) is set to 1 to balance the loss and it indicates that the bridge is implemented on both the generator and the discriminator.

**UAN**: the backbone or feature extractor is an ImageNet pretrained ResNet-50 [65] following what was done in the original paper. The classifier is a 2-layer fully connected network where the final output layer size depends on the dataset. Both the adversarial and non-adversarial discriminators are 3 layer FC networks with sigmoid activation function applied to the final output. While training, the learning rates for both the discriminators and the classifier was set as 0.001 and that of the feature extractor was set as 0.0001. Training was done with mini-batch SGD optimizer with momentum of 0.9.

**SHOT**: in these experiments, the network architecture is the same as described in their Github implementation. The backbone is a pretrained ResNet-50 [65] where the classifier layer is replaced with an FC layer of 256 neurons followed by a BN layer. The classifier layer consists of an FC layer followed by a weight normalization (WN) layer as shown in Figure 4. We consider a base learning rate of $\eta_{0}=10^{-2}$ for the backbone and 10 times the learning rate of the upper level layers. Similar to the original implementation, we also use the SGD optimizer with 0.9 momentum. We have used a learning rate scheduler $\eta =\eta_{0}\cdot {\left(1+10\cdot p\right)}^{-0.75}$ where *p* is the training process from 0 to 1. We have also set the hyperparameter β=0.3.

**SRDC**: The backbone network is a ResNet-50 [65] which is pretrained on ImageNet, as done in the original paper. In the backbone network the last FC layer is replaced with the task specific FC layer to parameterize the classifier. The pretrained layers are fine tuned, and the new layers are trained where the learning rate of the new layers is 10 times that of the pre-trained layers. For the training process mini-batch SGD is used with the momentum of 0.9 and a weight decay of 0.0001. The batch size is taken as 16. For each run, the best performing clustering model is used as the test model. Additional regularization is done by performing discriminative clustering in the bottleneck feature space.

## 6. Results and Discussion

In this section, we compare the performance using the overall accuracy and F1 scores of the different DA models on the aerial datasets. We also report the confusion matrix and t-SNE plots for the xView to DOTA domain adaptation scenario.

### 6.1. Performance Comparison

Table 5, Table 6 and Table 7 present the performance comparison for all the benchmarking methods. Each of the methods was executed five times and the mean and standard deviation of the accuracy was reported. The F1 scores for the methods are also reported as the classes are not perfectly balanced. F1 score is the harmonic mean of the precision and recall. Precision is the measure of how many positive predictions are correct. It is defined as the ratio of true positives over the sum of true positives and false positives. Recall is the measure of the correct positive cases from all the actual positive cases. It is defined as the ratio of the true positives over the sum of the true positives and false negatives. Every method except UAN achieved impressive performance for most of the datasets. For AID to UCM, SymNets acquires the highest performance gain with the classification accuracy jumped from 81.75% (before adaptation) to 99.15% (after adaptation). The F1 scores follow a similar trend as the accuracy scores except for the DOTA to xView adaptation. This may be because training on a higher resolution dataset, such as DOTA, and then adapting to a lower resolution one, like xView, is more challenging and leads to a degradation in performance. While being source free during adaptation, SHOT achieved competitive performance with the other source dependent methods.

### 6.2. xView to DOTA Results

**Figure 8:**
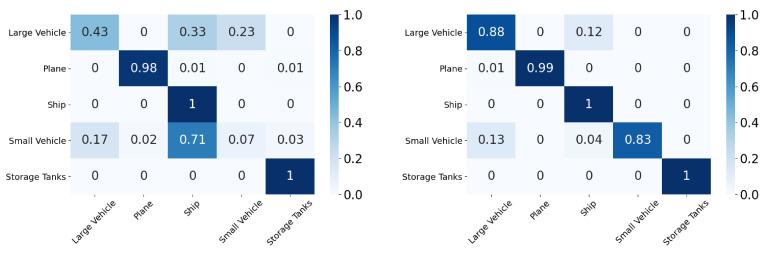
SymNets results for xView-DOTA dataset, before adaptation (**left**) and after adaptation (**right**).

**Figure 9:**
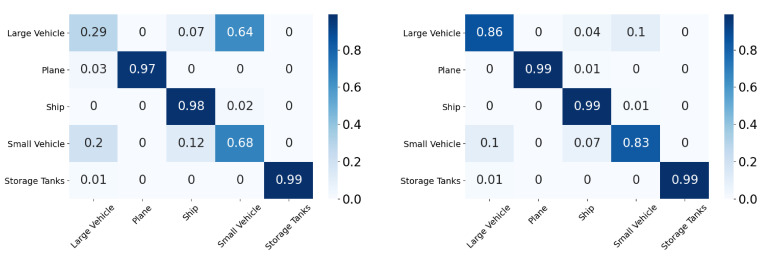
RSDA results for xView-DOTA dataset, before adaptation (**left**) and after adaptation (**right**).

**Figure 10:**
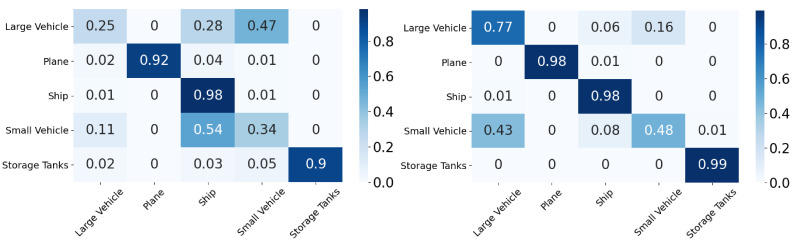
CDAN-GD results for xView-DOTA dataset, before adaptation (**left**) and after adaptation (**right**).

**Figure 11:**
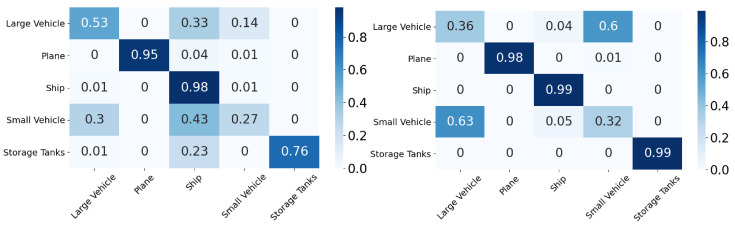
GVB-GD results for xView-DOTA dataset, before adaptation (**left**) and after adaptation (**right**).

**Figure 12:**
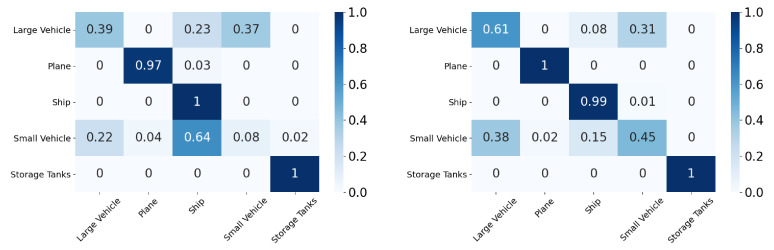
UAN results for xView-DOTA dataset, before adaptation (**left**) and after adaptation (**right**).

**Figure 13:**
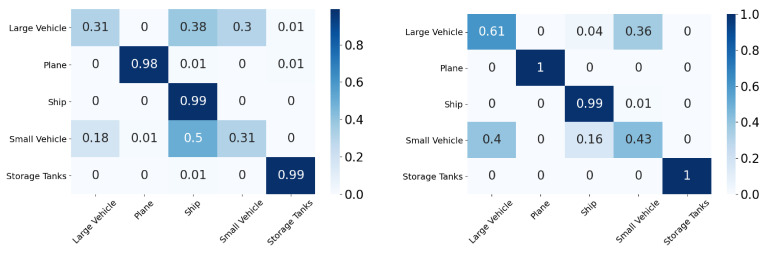
SHOT results for xView-DOTA dataset, before adaptation (**left**) and after adaptation (**right**).

**Figure 14:**
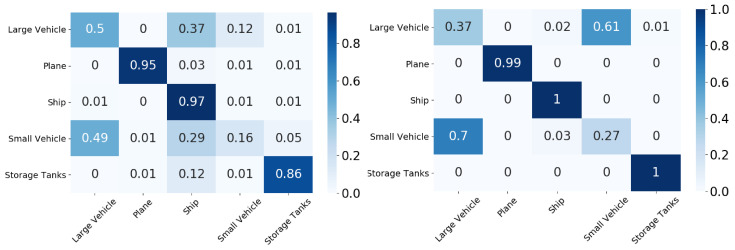
SRDC results for xView-DOTA dataset, before adaptation (**left**) and after adaptation (**right**).

**Figure 15:**
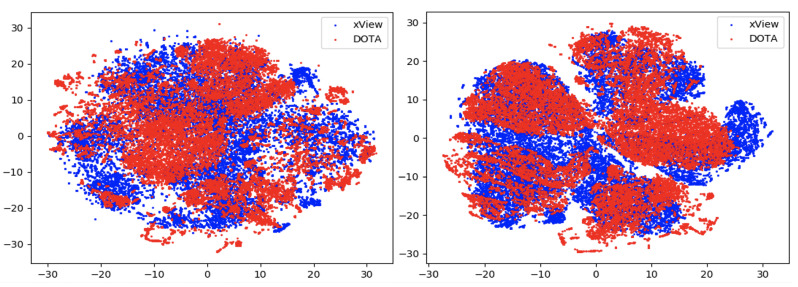
SymNets t-SNE for xView-DOTA dataset, before adaptation (**left**) and after adaptation (**right**). Blue points correspond to source domain (xView), and red points to target domain (DOTA).

**Figure 16:**
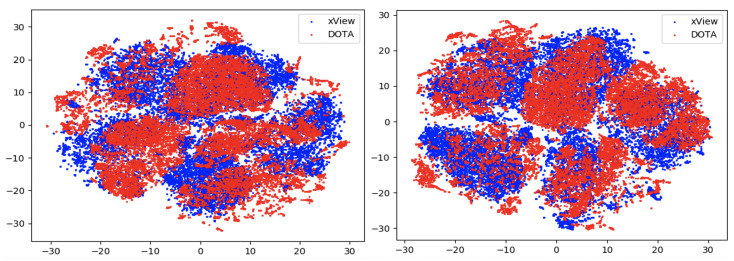
RSDA t-SNE for xView-DOTA dataset, before adaptation (**left**) and after adaptation (**right**). Blue points correspond to source domain (xView), and red points to target domain (DOTA).

**Figure 17:**
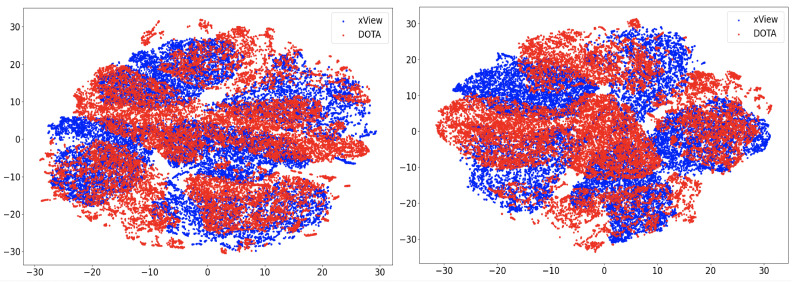
CDAN-GD t-SNE for xView-DOTA dataset, before adaptation (**left**) and after adaptation (**right**). Blue points correspond to source domain (xView), and red points to target domain (DOTA).

**Figure 18:**
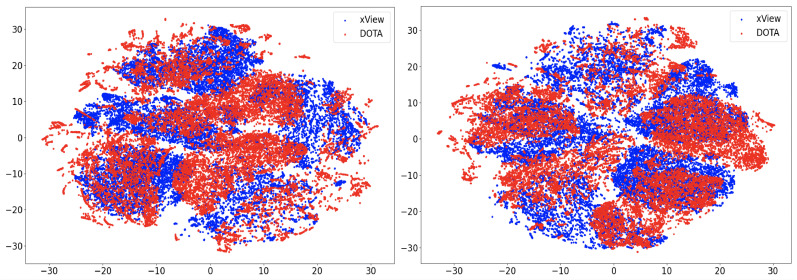
GVB-GD t-SNE for xView-DOTA dataset, before adaptation (**left**) and after adaptation (**right**). Blue points correspond to source domain (xView), and red points to target domain (DOTA).

**Figure 19:**
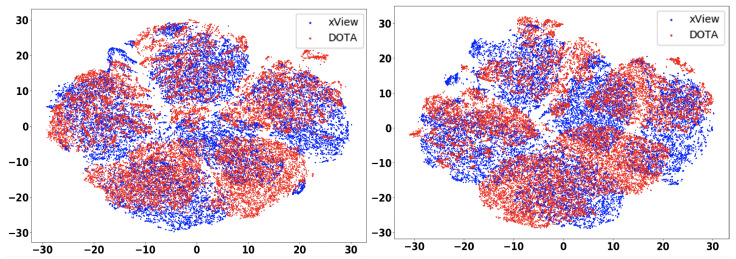
UAN t-SNE for xView-DOTA dataset, before adaptation (**left**) and after adaptation (**right**). Blue points correspond to source domain (xView), and red points to target domain (DOTA).

**Figure 20:**
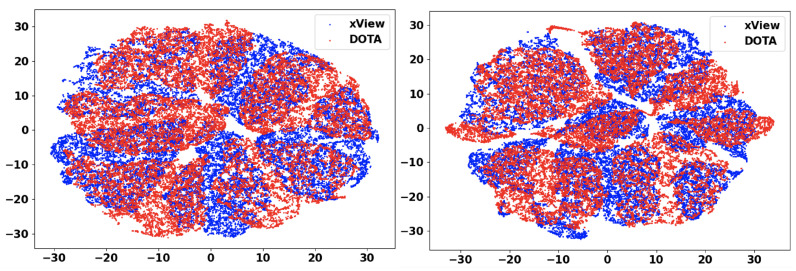
SHOT t-SNE for xView-DOTA dataset, before adaptation (**left**) and after adaptation (**right**). Blue points correspond to source domain (xView), and red points to target domain (DOTA).

**Figure 21:**
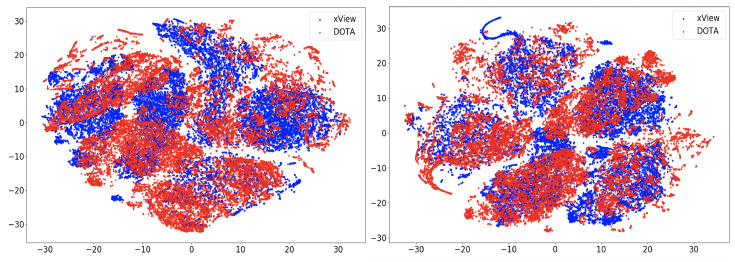
SRDC t-SNE for xView-DOTA dataset, before adaptation (**left**) and after adaptation (**right**). Blue points correspond to source domain (xView), and red points to target domain (DOTA).

In this section, we will discuss the results of the xView to DOTA adaptation in more detail with the help of confusion matrices (Figure 8, Figure 9, Figure 10, Figure 11, Figure 12, Figure 13 and Figure 14) and t-SNE plots (Figure 15, Figure 16, Figure 17, Figure 18, Figure 19, Figure 20 and Figure 21) of the various DA methods before and after adaptation. The custom xView to DOTA dataset was chosen for further analysis as there is a significant jump in accuracy from before DA to after DA for all the methods in this study, with SymNets showing the highest performance gain. A confusion matrix is used to quantify the performance of a classifier, where each row corresponds to the actual class and each column corresponds to the predicted class. It is used to show which classes are confused with each other for a given model, where ideal performance results in a diagonal matrix.

While there is an improvement in the performance for all classes across all DA methods, the most confusion appears to be between small vehicle and large vehicle or between ship and storage tank. This could be due to the fact that at small resolutions, small and large vehicles appear similar as do ships and storage tanks.

### 6.3. Feature Visualization

To visualize the features, we make use of t-Distributed Stochastic Neighbor Embedding (t-SNE) [26], which is a dimensionality reduction method used to visualize high-dimensional data on a 2D or 3D plot. t-SNE works in three steps: first, the similarity between points in the higher dimensional-space is measured. Next, a distribution that measures the pairwise distances between points in the lower-dimensional embedding is calculated. Finally, KL divergence is used to minimize the difference between the probability distributions in the higher and lower dimensional spaces to provide the final 2D graph for visualization.

To visualize features in this benchmarking study, we show t-SNE plots with the source and target domain features of the network before and after adaptation. Prior to the adaptation process, the t-SNE plot is obtained when the model is trained only on the source domain. The t-SNE plot after adaptation is used to visualize the improvement in the alignment of source and target domain features after the model is adapted to the target domain. In this section, we show the t-SNE plots for all the DA methods in Figure 15, Figure 16, Figure 17, Figure 18, Figure 19, Figure 20 and Figure 21, before and after adaptation on the xView-DOTA dataset which contains five classes, as outlined in Table 3.

When the domain adaptation process is successful, the source and target domains have near perfect alignment. From the t-SNE plots of the DA methods, SymNets, RSDA and SHOT seem to have the best source and target domain alignment after adaptation, as seen in Figure 15, Figure 16 and Figure 20.

## 7. Conclusions

We presented a benchmarking study of seven unsupervised domain adaptation methods (SHOT, RSDA, Symnet, UAN, GVB-GD, CDAN-GD, and SRDC) on three custom aerial DA datasets. These datasets were created by taking the common classes between AID-UCM, DOTA-xView, and NWPU-CLRS. We reported the accuracy for each of the methods considered on the aerial datasets to determine their efficacy. The confusion matrices and t-SNE plots of the methods on the xView-DOTA dataset were also reported.

For the AID to UCM adaptation, SymNets does the best, while SHOT reports the most improvement for the UCM to AID adaptation task. For CLRS to NWPU and NWPU to CLRS adaptation, SHOT shows the best performance. Finally, for the DOTA to xView dataset, SHOT has highest accuracy, while SymNets does best for the xView to DOTA adaptation. Overall, SHOT and SymNets are the best performing models for the task of aerial domain adaptation. This is further evidenced by observing the confusion matrix of SymNets for the xView to DOTA adaptation, where after adaptation, the diagonal gets stronger. The t-SNE plots of SymNets and SHOT both show good alignment of the source and target domains after adaptation, which is further evidence of the efficacy of these methods.

## Figures and Tables

**Table 1:** AID-UCM DA Dataset.

AID Classes	Number of Samples	UCM Classes	Number of Samples
Airport	360	Airplane	100
Parking	390	Parking Lot	100
Storage Tank	360	Storage Tank	100
Beach	400	Beach	100
Forest	350	Forest	100
River	410	River	100
Baseball Field	220	Baseball Diamond	100
Medium Residential	290	Medium Residential	100
Sparse Residential	300	Sparse Residential	100

**Table 2:** NWPU-CLRS DA Dataset.

NWPU Classes	Number of Samples	CLRS Classes	Number of Samples
Airplane	700	Airplane	600
Bridge	700	Bridge	600
Parking	700	Parking	600
Railway Station	700	Parking	600
Runway	700	Parking	600
Storage Tank	700	Storage Tank	600

**Table 3:** DOTA-xView DA Dataset.

DOTA Classes	Number of Samples	Augmented Samples	xView Classes	Number of Samples	Augmented Samples
Large Vehicle	5000	0	Large Vehicle	5000	0
Plane	5000	0	Plane	1159	3841
Ship	5000	0	Ship	4476	524
Small Vehicle	5000	0	Small Vehicle	5000	0
Storage Tank	2126	2874	Storage Tank	1447	3553

**Table 4:** URLs for codes of implemented methods. All code is implemented in PyTorch.

Method	Base Network	Code Repository
SymNets	ResNet-50	https://github.com/YBZh/SymNets (accessed on 1 March 2021)
RSDA	ResNet-50	https://github.com/XJTU-XGU/RSDA (accessed on 1 March 2021)
CDAN-GD	ResNet-50	https://github.com/cuishuhao/GVB/tree/master/CDAN-GD (accessed on 1 March 2021)
GVB-GD	ResNet-50	https://github.com/cuishuhao/GVB/tree/master/GVB-GD (accessed on 1 March 2021)
UAN	ResNet-50	https://github.com/thuml/Universal-Domain-Adaptation (accessed on 1 November 2021)
SHOT	ResNet-50	https://github.com/tim-learn/SHOT (accessed on 1 March 2021)
SRDC	ResNet-50	https://github.com/huitangtang/SRDC-CVPR2020 (accessed on 1 March 2021)

**Table 5:** Accuracy and F1 scores comparison of DA Methods before adaptation (Before) and after adaptation (After) for AID-UCM DA dataset with the best performance in bold.

Method	AID→UCM				UCM→AID			
	Accuracy		F1 Score		Accuracy		F1 Score	
	Before	After	Before	After	Before	After	Before	After
SymNets	81.75 ± 1.25	**99.15 ± 0.22**	0.83	0.98	75.85 ± 1.03	98.37 ± 0.11	0.79	0.97
RSDA	**87.44 ± 1.62**	98.67 ± 0.26	**0.87**	**0.99**	**82.76 ± 0.85**	98.15 ± 0.12	**0.85**	**0.98**
CDAN-GD	84.53 ± 1.49	97.37 ± 0.26	0.79	0.97	80.43± 1.21	97.20 ± 0.27	0.8	0.97
GVB-GD	84.39 ± 1.02	97.63 ± 0.26	0.84	0.96	81.8 ± 0.40	97.41 ± 0.18	0.79	0.97
UAN	82.44 ± 0.01	84.24 ± 0.32	0.79	0.81	78.41 ± 0.46	85.34 ± 0.08	0.76	0.81
SHOT	83.28 ± 0.80	98.80 ± 0.26	0.80	**0.99**	77.38 ± 0.78	**98.55 ± 0.14**	0.76	**0.98**
SRDC	84.97 ± 0.14	95.57 ± 0.56	0.82	0.96	79.91± 0.94	95.89 ± 0.79	0.76	0.97

**Table 6:** Accuracy and F1 scores comparison of DA Methods before adaptation (Before) and after adaptation (After) for NWPU-CLRS DA dataset with the best performance in bold.

Method	CLRS→NWPU				NWPU→CLRS			
	Accuracy		F1 Score		Accuracy		F1 Score	
	Before	After	Before	After	Before	After	Before	After
SymNets	94.42 ± 0.51	98.11 ± 0.17	0.94	0.98	87.81 ± 0.69	95.51 ± 0.43	0.89	0.95
RSDA	94.26 ± 0.66	97.66 ± 0.13	0.87	**0.99**	89.00 ± 0.53	94.61 ± 0.27	0.89	0.93
CDAN-GD	95.57 ± 0.21	97.14 ± 0.21	0.96	0.98	88.34 ± 1.09	94.51 ± 0.77	0.86	0.95
GVB-GD	**96.19 ± 0.36**	97.16 ± 0.33	**0.97**	0.98	**89.25 ± 1.67**	93.86 ± 0.81	**0.91**	0.94
UAN	94.29 ± 0.23	91.79 ± 0.11	0.93	0.91	84.48 ± 0.28	90.63 ± 0.10	0.84	0.90
SHOT	94.41 ± 0.25	**98.27 ± 0.13**	0.94	0.98	87.49 ± 0.39	**96.14 ± 0.13**	0.88	**0.96**
SRDC	94.59 ± 0.50	97.68 ± 0.18	0.95	0.98	87.31 ± 0.64	93.51 ± 0.54	0.87	0.93

**Table 7:** Accuracy and F1 scores comparison of DA Methods before adaptation (Before) and after adaptation (After) for DOTA-xView DA dataset with the best performance in bold.

Method	DOTA→xView				xView→DOTA			
	Accuracy		F1 Score		Accuracy		F1 Score	
	Before	After	Before	After	Before	After	Before	After
SymNets	65.67 ± 0.52	70.82 ± 0.85	0.66	0.72	69.44 ± 0.56	**95.88 ± 0.76**	0.68	**0.96**
RSDA	64.33 ± 1.19	68.44 ± 1.28	0.66	0.71	**77.07 ± 1.18**	87.93 ± 4.22	**0.77**	0.93
CDAN-GD	64.74 ± 2.07	75.21 ± 1.22	0.64	0.75	71.84 ± 2.38	83.17 ± 3.28	0.66	0.84
GVB-GD	64.43 ± 2.07	73.41 ± 2.04	0.61	**0.76**	70.25 ± 1.44	84.84 ± 2.17	0.69	0.73
UAN	62.74 ± 0.17	68.13 ± 0.43	0.61	0.66	68.71 ± 0.22	80.78 ± 0.40	0.68	0.79
SHOT	**67.86 ± 0.38**	**75.36 ± 0.69**	0.67	0.73	71.19 ± 1.37	80.46 ± 2.37	0.69	0.79
SRDC	66.44 ± 0.36	70.50 ± 1.39	**0.70**	0.72	72.39 ± 2.14	84.88 ± 7.13	0.66	0.72
